# The Single Nucleotide Polymorphism *PPARG2* Pro12Ala Affects Body Mass Index, Fat Mass, and Blood Pressure in Severely Obese Patients

**DOI:** 10.1155/2018/2743081

**Published:** 2018-12-12

**Authors:** Ana Paula dos Santos Rodrigues, Lorena Pereira Souza Rosa, Hugo Delleon da Silva, Elisângela de Paula Silveira-Lacerda, Erika Aparecida Silveira

**Affiliations:** ^1^Health Science Post-Graduation Program, Faculty of Medicine, Universidade Federal de Goiás, 1a Avenida s/n, Setor Universitário, Goiânia, Goiás, Brazil; ^2^Institute of Science and Technology (FIBRA), BR 060-153 KM 97 N° 3400, São João, Anápolis, GO 75000-001, Brazil; ^3^Uni-Anhanguera University Center of Goias, Av. João Candido de Oliveira, 115-Cidade Jardim, Goiânia, GO 74423-115, Brazil; ^4^Molecular Biology and Cytogenetics Laboratory, Institute of Biological Sciences, Universidade Federal de Goiás, Av. Esperança, s/n, Campus Samambaia (Campus II), Goiânia, Goiás, Brazil

## Abstract

**Background:**

The *PPARG2* Pro12Ala (rs1801282) and *IL6* -174G >C (rs1800795) have important function in body weight regulation and a potential role in obesity risk. We aimed to investigate the association between *PPARG2* Pro12Ala and *IL6* -174G >C variants and the genotypes interaction with body composition, metabolic markers, food consumption, and physical activity in severely obese patients.

**Methods:**

150 severely obese patients (body mass index (BMI) ≥ 35 kg/m^2^) from Central Brazil were recruited. Body composition, metabolic parameters, physical activity, and dietary intake were measured. The genotype was determined by the qPCR TaqMan Assays System. Multiple linear regression and multiple logistic regression models were fitted adjusting for confounders.

**Results:**

Ala carriers of the Pro12Ala polymorphism had higher adiposity measures (BMI: *p*=0.031, and fat mass: *p*=0.049) and systolic blood pressure (*p*=0.026) compared to Pro homozygotes. We found no important associations between the -174G >C polymorphism and obesity phenotypes. When genotypes were combined, individuals with genotypes ProAla + AlaAla and GC + CC presented higher BMI (*p*=0.029) and higher polyunsaturated fatty acids (PUFAs) consumption (*p*=0.045) compared to the ones with genotypes ProPro and GG, and individuals carriers of the *PPARG2* Ala allele only (genotype ProAla + AlaAla and GG) had higher fat mass and systolic and diastolic blood pressure compared to the ones with genotypes ProPro and GG.

**Conclusions:**

Severely obese individuals carrying the Ala allele of the *PPARG2* Pro12Ala polymorphism had higher measures of adiposity and blood pressure, while no important associations were found for the *IL6* -174G >C polymorphism.

## 1. Introduction

Studies about the genetic obesity susceptibility have investigated polymorphisms related to genes encoding factors involved in food and energy intake regulation, energy expenditure, and adipogenesis control [1]. In this context, the polymorphisms *PPARG2* Pro12Ala (rs1801282) and *IL6* -174G >C (rs1800795) have received attention for their possible influence in body weight regulation [1–3].

The *PPARG2* has an important role in modulating the expression of genes involved in the regulation of adipose tissue differentiation and lipid metabolism [4, 5]. The mostly studied polymorphism of *PPARG2*, the Pro12Ala, may promote lower affinity of the PPAR-*γ* for the response element and about 50% lower transcriptional capacity [6]. The Ala allele has been associated with lower body mass index (BMI) [6, 7]; nevertheless, some meta-analyses have found contradictory results showing higher BMI in Ala carriers, especially in severely obese individuals [8–11]. Considering this potential association of the Ala allele and increased BMI, it is important to investigate whether this variant influences other parameters in severely obese individuals.

Regarding interleukin-6 (IL-6), one of the major proinflammatory cytokines, it is positively related to increased BMI [3, 12]. The association studies between obesity and the *IL6* -174G >C polymorphism have shown higher BMI in the presence of C allele in cross-sectional and cohort studies [13–17], but it was not confirmed by meta-analysis [18, 19]. Thus, the role of Pro12Ala and -174G >C in modulating BMI is still to be confirmed and also the information regarding other body composition parameters such as fat mass, fat-free mass, percentage body fat, and lean mass.

Interaction of genes with environmental factors, such as diet and physical activity, may be involved in the discrepancies of associations between studies [1, 20, 21]. Gene-diet interaction studies with Pro12Ala and -174G >C polymorphisms have found that the energy content and composition of the diet may affect obesity phenotypes, showing the importance to assess the interactions among genotypes and potentially modifiable lifestyle factors [20, 22, 23]. Considering that the *PPARG2* Pro12Ala and *IL6* -174G >C polymorphisms have important functions in body weight regulation with a potential role in obesity risk and the alarming increase in severe obesity worldwide [24], they are promising single nucleotide polymorphisms (SNPs) for association studies of obesity phenotypes. Thus, we aimed to investigate the association between *PPARG2* Pro12Ala and *IL6* -174G >C variants and the genotypes interaction with body composition, metabolic markers, food consumption, and physical activity in severely obese patients.

## 2. Methods

### 2.1. Subjects

This study is an analysis of baseline data from participants of the clinical trial “Effect of Nutritional Intervention and Olive Oil in Severe Obesity: Randomized Controlled Trial” (DieTBra Trial) (registered at ClinicalTrials.gov: NCT02463435). A total of 150 severely obese patients (BMI ≥ 35 kg/m^2^) aged 18 to 65 years were recruited from primary care of the Brazilian Unified Health System at Goiânia, Goiás State, in Central Brazil. Patients were referred to the Nutrition in Severe Obesity Outpatient Clinic, and the study took place at the Clinical Research Unit of the Clinical Hospital/Federal University of Goiás. The study excluded individuals that had already underwent bariatric surgery, under actual nutritional treatment for weight loss or in the previous 2 years, using antiobesity or anti-inflammatory drugs, having HIV/AIDS, as well as heart/kidney/hepatic insufficiency, chronic obstructive pulmonary disease, cancer, and pregnancy. Patients were recruited from June to November 2015. All patients who agreed to participate in the study gave written consent. The study was approved by the Research Ethics Committee of Clinical Hospital of Federal University of Goiás (protocol number 747.792).

### 2.2. Anthropometric and Body Composition Measurements

Body weight and height were measured using standardized procedures [25]. BMI (kg/m^2^) was calculated dividing the body mass (kg) by the squared height (m^2^). Severe obesity was defined as BMI ≥ 35 kg/m^2^ [24].

Fat mass (kg), fat-free mass (kg), percentage body fat, and lean mass were measured using multifrequency bioelectrical impedance analysis (BIA). The measurement was performed with the InBody S10 device (Biospace Co., Ltd., Seoul, Korea) by using different frequencies (1, 5, 50, 250, 500, and 1000 kHz) at each segment (right arm, left arm, trunk, right leg, and left leg).

For BIA assessment, patients were instructed to fast for 12 h and avoid strenuous physical activity and alcohol, as well as food and drinks containing caffeine on the previous day [26]. BIA assessment was conducted according to the manufacturer's guidelines.

### 2.3. Dietary Intake

Food consumption was assessed using three 24 h records collected within seven days, being two face to face and one by phone. Data were assessed by trained registered dieticians. We used the multiple pass method (MPM) to collect the 24 h records [27], and the nutritional analysis was performed using Avanutri Online® (Avanutri Equipamentos de Avaliação Ltda, Rio de Janeiro, BR). Energy (kcal), proteins (%), carbohydrates (%), lipids (%), saturated fatty acids (SAFs) (%), monounsaturated fatty acids (MUFAs) (%), polyunsaturated fatty acids (PUFAs) (%), polyunsaturated : saturated fatty acids ratio (P : S ratio), cholesterol (g), and fiber (g) were obtained calculating the mean of the three 24 h records.

### 2.4. Physical Activity Assessment

Physical activity was assessed using a triaxial accelerometer ActiGraph wGT3X (ActiGraph, Pensacola, FL, USA) for movement registration. Patients were instructed to wear the accelerometer 24 h a day for six consecutive days over the nondominant wrist, even during shower and water activities, as the device was waterproof. The sampling frequency of the accelerometer was set at 30 Hz, and the data collection interval was set at one min. Accelerometers were set up and downloaded at ActiLife 6 software. Output data were processed using the R-package GGIR (http://cran.r-project.org). The outcome measures used in the present study were moderate-to-vigorous physical activity (MVPA) (>100 mg) defined as estimated time spent in ≥ 10 min per bout during a week and the sedentary time (<50 mg, without bouts) in min per day.

### 2.5. Blood Pressure and Comorbidities

Systolic and diastolic blood pressures were measured with the patient in the sitting position after resting for at least 5 min. Two measures were taken within the 3 min interval using the Omron HEM 742INT (Omron Healthcare Inc., Kyoto, Japan) automatic blood pressure monitor with an appropriately sized cuff, and the mean was calculated.

The presence of comorbidities was analyzed as a dichotomous variable (presence/absence). Subjects with systolic/diastolic blood pressure higher than 140/90 mmHg or under antihypertensive therapy were considered hypertensive [28]. Subjects with fasting glucose ≥ 126 mg/dL or under glucose-lowering therapy were considered diabetic [29]. Individuals with low-density lipoprotein (LDL) cholesterol ≥ 160 mg/dL and/or triglycerides ≥ 150 mg/dL or high-density lipoprotein (HDL) cholesterol < 40 mg/dL for men and < 50 mg/dL for women were classified as dyslipidaemic [30].

### 2.6. Laboratory Tests

Blood samples were collected for metabolic markers and genomic DNA extraction after 12 h overnight fasting. Serum glucose, total cholesterol, HDL cholesterol, LDL cholesterol, and triglycerides were measured by enzyme-colorimetric methods. Serum insulin was measured by chemiluminescence, and hemoglobin A1c (HbA1c) was measured by liquid chromatography. The homeostasis model assessment of insulin resistance (HOMA-IR) was calculated following the formula derived by Matthews et al. [31].

### 2.7. DNA Extraction and Genotyping

Genomic DNA was extracted from whole blood with the PureLink™ Genomic DNA Mini Kit (Invitrogen, Carlsbad, CA, USA). DNA concentration and purity were evaluated by spectrophotometric determination of the *A*_260/280_ ratio with NanoDrop® 2000c (Thermo Fisher Scientific, Waltham, MA, USA), and DNA quality was checked using agarose gel electrophoresis. Genotyping was performed using custom TaqMan SNP genotyping assays—ID c__1129864_10 for *PPARG2* Pro12Ala (rs1801282) and ID c__1839697_20 for *IL6* -174G >C (rs1800795) (Applied Biosystems, Foster City, CA, USA)—on a StepOnePlus™ Real-Time PCR System (Thermo Fisher Scientific, Waltham, MA, USA). The standard real-time polymerase chain reaction (qPCR) was carried out using the TaqMan GTXpress™ Master Mix (Thermo Fisher Scientific, Waltham, MA, USA) reagent kit in a 21 *µ*L volume according to the manufacturer's instructions. Although DNA samples were extracted for all study participants, the qPCR amplification was only conducted for the *PPARG2* Pro12Ala polymorphism on samples from 146 individuals and for the *IL6* -174G >C polymorphism on samples from 148 individuals.

### 2.8. Statistical Analysis

The dataset was structured using EpiData 3.1, and double entry typing with validation was performed. The data were presented as mean ± SD for continuous variables and frequencies for categorical variables. The chi-squared test was used to analyze the agreement of genotype frequencies with the Hardy–Weinberg equilibrium expectation. Allele frequency was determined by manual counting. Normal distribution was tested for all measured variables using the Kolmogorov–Smirnov test and histograms; skewed variables were normalized by log transformation and then backtransformed for results presentation.

Individual genotype analysis and the combination of the two genotypes analysis (*PPARG2* + *IL6*) were performed. For the combination of genotypes, patients were grouped as follows: no variants (genotypes ProPro and GG, *n*=78), *IL6* only (genotypes ProPro and GC + CC, *n*=48), *PPARG2* only (genotypes ProAla + AlaAla and GG, *n*=15), and both variants (genotypes ProAla + AlaAla and GC + CC, *n*=4). Comparisons were performed using Student's *t*-test or ANOVA and chi-squared test or Fisher's exact test. Due to the low frequency of the variant allele, we compared carriers versus noncarriers of the minor allele. We fitted multiple linear regression models to adjust the analysis for potential confounders (age, sex, BMI, sedentary time, and diabetes). For binary variables, odds ratios (ORs) and 95% confidence intervals (95% CIs) were calculated and multiple logistic regression models were fitted adjusting for the same confounders. Statistical analyses were performed in Stata 12.

## 3. Results

All the study participants (*n*=150) had DNA extracted; however, genetic samples were viable in 146 individuals for the *PPARG2* Pro12Ala polymorphism and in 148 individuals for the *IL6* -174G >C polymorphism. The genotype distribution for *PPARG2* Pro12Ala was 86.9%, 12.4%, and 0.7% for ProPro, ProAla, and AlaAla, respectively. The minor allele frequency of the Ala allele was 0.065. For *IL6* -174G >C, the frequencies of GG, GC, and CC genotypes were 65.3%, 31.3%, and 3.4%, respectively. The minor allele frequency of the C allele was 0.193. Observed genotype frequencies were in agreement with the Hardy–Weinberg equilibrium (*p*=0.689 for *PPARG2* Pro12Ala and *p*=0.863 for *IL6* -174G >C).

The characteristics of the study participants according to the *PPARG2* Pro12Ala polymorphism are displayed in Table 1. Analysis of the Pro12Ala polymorphism showed higher BMI (*p*=0.031) and fat mass (*p*=0.049) for Ala carriers, even after adjustment for age, sex, sedentary time, and diabetes. Ala carriers presented significantly higher SBP and DBP, but after adjustments, only SBP (*p*=0.026) remained associated (Table 1).

The characteristics of the study participants according to the *IL6* -174G >C polymorphism are displayed in Table 2. Sex and MVPA were associated with the -174G >C polymorphism after adjustments (*p*=0.043 and *p*=0.024, respectively). Males had triple probability (OR: 3.60; 95% CI: 1.04–12.48) to be C carriers, and the C carriers spent lower amount of time in MVPA (Table 2).

**Figure 1:**
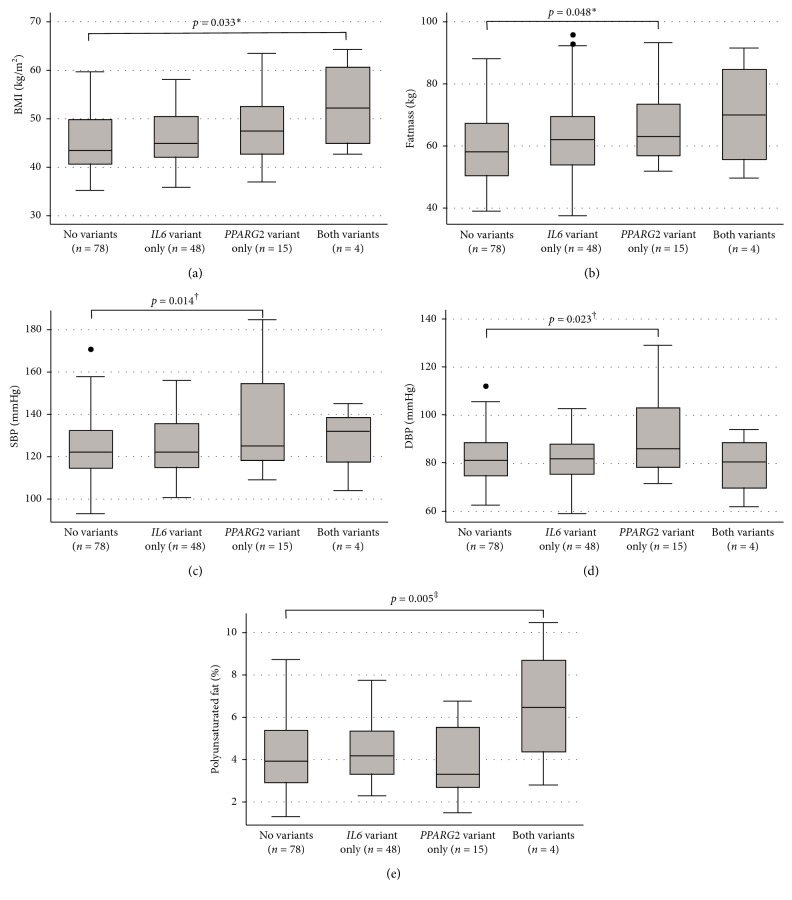
Comparison of BMI (a), fat mass (b), SBP (c), DBP (d), and percentage of polyunsaturated fat consumption (e) between different genotype combinations in severely obese patients. BMI: body mass index; SBP: systolic blood pressure; DBP: diastolic blood pressure. Results are expressed as median (percentiles 25–75%). Dots represent outlier values. ^*∗*^Adjusted for age, gender, and sedentary time. ^†^Adjusted for age, gender, sedentary time, BMI, and diabetes. ^‡^Adjusted for age, gender, BMI, and diabetes.

For combined genotypes, participants were grouped as follows: no variants (*n*=78), *IL6* variant only (*n*=48), *PPARG2* variant only (*n*=15), and both variants (*n*=4). The same variables of the individual genotype analysis were tested for the combined genotypes, but the results were presented in figures only for the variables with a significant statistical difference. Analysis of the combined effects of the two genotypes showed association with BMI, fat mass, SBP, DBP, and polyunsaturated fat consumption after adjustments. Individuals with both variants had higher BMI (*p*=0.023) compared to the ones with no variants. Fat mass, SBP, and DBP were higher for participants with the *PPARG2* variant only compared to those with no variants (*p*=0.045, *p*=0.018, and *p*=0.030, respectively). Individuals with both variants presented higher consumption of PUFA compared to the ones with no variants (*p*=0.045) (Figure 1). The analysis of BMI in categories did not show association with the polymorphisms or the genotype combination (Table 3).

## 4. Discussion

The Ala allele of *PPARG2* Pro12Ala (rs1801282) is potentially associated with higher BMI; however, few studies have investigated other key factors that may interact with the variant leading to severe obesity susceptibility such as metabolic, food consumption, and clinical parameters. To our knowledge, this is the first study to find association of the *PPARG2* Pro12Ala (rs1801282) polymorphism with an obesity phenotype other than BMI and blood pressure in severely obese patients. We found higher measures of adiposity (BMI and fat mass) and higher SBP in Ala carriers of the Pro12Ala polymorphism compared to the ProPro individuals. These results may contribute to a better understanding of the pathophysiology of severe obesity and translate into more effective preventive and treatment measures to halt its increasing prevalence.

For the *PPARG2* Pro12Ala polymorphism, we observed a genotype distribution (87% for ProPro and 13% for Ala carriers) similar to that in the few studies with Brazilians [32, 33]. Among studies with morbidly obese individuals (BMI ≥ 40 kg/m^2^), frequencies range from 72.5% to 86.0% for the ProPro genotype and from 14.0% to 27.5% for Ala carriers [34–38]. For the -174G >C polymorphism, frequencies vary widely. In the current study, we observed 64.9% of GG and 35.1% of GC + CC, while French morbidly obese individuals presented frequencies of 43.1% for GG and 56.9% for GC + CC [39].

Despite controversial results regarding the *PPARG2* Pro12Ala polymorphism, meta-analyses have shown association of the Ala allele with higher BMI and fat mass, especially in severely obese individuals, corroborating our results [6–8, 10, 11, 40]. The controversies observed in other studies may be explained by a suggested interaction between dietary fat intake and the Pro12Ala polymorphism, showing that when the dietary P : S ratio is similar or lower than 0.66, Ala carriers present higher BMI than Pro homozygotes, while when the P : S ratio is higher than 0.66, the opposite occurs [41]. Our patients had the mean P : S ratio ≤ 0.66, possibly explaining the higher BMI among Ala carriers and demonstrating the effect of dietary fat intake on the phenotype determination.

PPAR-*γ*2 has a crucial role in modulating lipid metabolism and adipose tissue accumulation [4, 5]. The *PPARG2* Pro12Ala polymorphism has been suggested to have a role in the variance of fat mass among analyses of candidate genes for adiposity changes [42]. Few studies have investigated the influence of the Pro12Ala polymorphism on body composition parameters other than body mass, BMI, and waist circumference. Association between the Ala allele and higher fat mass was found in Caucasians from the Québec Family Study [43] and in Italian women [44], corroborating the results of the current study. Thus, more detailed measurements of body composition are important to be addressed in studies of the Pro12Ala polymorphism.

We found an association between the Ala allele of the Pro12Ala polymorphism and higher blood pressure in severely obese individuals. Although the association between the Pro12Ala variants and blood pressure is controversial, a meta-analysis of eight studies with 3281 individuals (1865 cases and 1416 controls) suggested that the Ala allele might be protective for hypertension among East Asians, but not among Caucasians [45]. The mechanisms behind this association are unclear. Studies have suggested that the activation of the PPAR-*γ*2 inhibits processes related to inflammation and hypertension. The Ala allele has been associated with impaired function of the PPAR-*γ*2, leading to increased blood pressure [46–48].

We found higher probability of severely obese males to be C carriers of the *IL6* -174G >C polymorphism after adjustments. Studies comparing elderly (octa/nonagenarians and centenarians) with younger individuals have found a tendency in the reduction of GG genotypes in males [49–52]. Regarding MVPA, we did not find any evidence that justifies this association. More investigation is needed to clarify the association of the higher frequency of C alleles in severely obese males and the -174G >C polymorphism and also the association of the C allele with lower time spent in MVPA.

When the Pro12Ala and the -174G >C genotypes were combined, association was found between the *PPARG2* variant only and fat mass, SBP, and DBP compared to the ones with no variants, as demonstrated in the independent analysis of genotypes. We also observed higher BMI and higher PUFA consumption in the presence of both variants compared to the ones with no variants. Nevertheless, this association of the combined genotypes seems to be determined by the *PPARG2* Pro12Ala polymorphism as the *IL6* -174G >C polymorphism was not associated with adiposity indexes in the independent analysis of genotypes and also due to the low number of individuals with both variants.

Our study has limitations such as the small sample size, especially in the analysis of combined genotypes, and the impossibility to demonstrate causality due to the study design. However, we may address some strengths, such as the analysis of the association between SNPs and also the combination of genotypes, with a variety of parameters, such as anthropometrical, clinical, biochemical, physical activity, and dietary intake, in severely obese patients; the adjusted analysis for potential confounders, such as sociodemographic, anthropometrical, and physical activity measurements; and the high-quality methods used to assess physical activity (triaxial accelerometer) and body composition (BIA).

## 5. Conclusions

In summary, we found that severely obese individuals carrying the Ala allele of the *PPARG2* Pro12Ala polymorphism have higher measures of adiposity (BMI and fat mass) and blood pressure, while no important associations were found for the *IL6* -174G >C polymorphism. Further studies on gene-diet/gene-environment interactions are necessary to clarify the associations and underlying mechanisms between SNPs and severe obesity. This information may eventually be used to develop tailored interventions specific to the individual's genotype and enable more effective prevention and treatment measures to address this increasing public health problem.

## Figures and Tables

**Table 1:** Demographic and clinical characteristics of studied participants according to the *PPARG2* Pro12Ala (rs1801282) polymorphism.

Variables	Total	Pro12Ala polymorphism		*p* value^*∗*^	Adjusted *p* value
	*N*=146	ProPro (*N*=127)	Ala carriers (*N*=19)		
*Clinical, anthropometrical, and body composition variables*					
Age (years)	39.82 ± 8.70	40.00 ± 8.64	38.63 ± 2.12	0.524	
Female/male, *N* (%)	125 (85.6)/21 (14.4)	108 (86.4)/19 (90.5)	17 (13.6)/2 (9.5)	1.000^†^	0.427^a^
BMI (kg/m^2^)	46.09 ± 6.42	45.62 ± 6.12	49.26 ± 1.75	**0.020**	**0.026** ^b^
Fat mass (kg)^1^	61.43 ± 13.02	60.52 ± 12.73	67.67 ± 13.67	**0.029**	**0.046** ^b^
Fat-free mass (kg)^1^	57.22 ± 8.86	57.00 ± 9.06	58.77 ± 7.43	0.431	0.227^b^
SBP (mmHg)	128.66 ± 17.89	124.35 ± 13.82	133.92 ± 22.51	**0.011**	**0.022** ^c^
DBP (mmHg)	85.92 ± 13.68	81.63 ± 9.65	87.60 ± 16.04	**0.024**	0.050^c^
Diabetes, *N* (%)	40 (27.4)	36 (28.4)	4 (21.0)	0.593^‡^	0.400^d^
Hypertension, *N* (%)	96 (65.8)	83 (65.4)	13 (68.4)	0.793^†^	0.760^c^
Dyslipidemia, *N* (%)	114 (78.1)	102 (80.3)	12 (63.2)	0.099^‡^	0.122^c^
Log MVPA (min/week)^2^	44.54 ± 61.42	46.67 ± 63.73^§^	31.17 ± 43.27^§^	0.260	0.204^e^
Sedentary time (min/day)^2^	1175.25 ± 83.06	1174.10 ± 83.45	1182.44 ± 80.56	0.685	0.817^e^

*Biochemical parameters*					
Fasting glucose (mg/dL)	110.73 ± 45.38	112.12 ± 47.92	101.47 ± 20.15	0.342	0.350^d^
Fasting glucose range, *N* (%)				0.596^†^	0.441^d^
<100 mg/dL, *N* (%)	85 (58.2)	75 (88.2)	10 (11.8)		
≥100 mg/dL, *N* (%)	29 (19.9)	52 (85.2)	9 (14.8)		
Fasting insulin (*μ*U/mL)	23.43 ± 14.88	23.04 ± 14.51	26.03 ± 17.34	0.415	0.413^d^
HOMA-IR	6.44 ± 4.91	6.42 ± 4.86	6.56 ± 4.72	0.915	0.829^d^
GHb (%)	6.29 ± 1.45	6.30 ± 1.47	6.23 ± 1.32	0.843	0.875^d^
Total cholesterol (mg/dL)	188.68 ± 36.57	190.42 ± 36.86	177.05 ± 33.12	0.138	0.232^c^
HDL cholesterol (mg/dL)	47.47 ± 11.11	47.69 ± 11.49	45.95 ± 8.20	0.525	0.501^c^
LDL cholesterol (mg/dL)	119.20 ± 34.01	110.08 ± 34.68	103.42 ± 29.42	0.429	0.497^c^
Triglyceride (mg/dL)	160.16 ± 78.60	163.42 ± 80.67	138.37 ± 60.34	0.196	0.331^c^

*Dietary intake*					
Energy (kcal)	1709.50 ± 704.50	1682.55 ± 695.05	1889.62 ± 759.68	0.233	0.139^e^
Proteins (%)	17.42 ± 4.60	17.58 ± 4.61	16.34 ± 4.54	0.274	0.239^e^
Carbohydrates (%)	51.64 ± 8.56	51.79 ± 8.72	50.64 ± 7.49	0.589	0.593^e^
Lipids (%)	27.97 ± 6.59	27.74 ± 6.70	29.51 ± 5.70	0.278	0.245^e^
Saturated (%)	8.38 ± 2.65	8.30 ± 2.70	8.98 ± 2.26	0.294	0.372^e^
Polyunsaturated (%)	4.29 ± 1.66	4.25 ± 1.57	4.52 ± 2.24	0.515	0.452^e^
Monounsaturated (%)	7.62 ± 2.60	7.54 ± 2.63	8.16 ±2.40	0.363	0.509^e^
P : S ratio	0.56 ± 0.28	0.56 ± 0.27	0.55 ± 0.36	0.438	0.475^e^
Cholesterol (g)	222.50 ± 120.74	217.38 ± 114.16	256.66 ± 157.45	0.187	0.206^e^
Fiber (g)	15.28 ± 8.04	15.03 ± 8.21	16.52 ± 9.72	0.472	0.452^e^

Data are presented as mean ± SD or *N* (%). BMI: body mass index; HOMA-IR: homeostatic model assessment for insulin resistance; GHb: glycated hemoglobin; HDL: high-density lipoprotein; LDL: low-density lipoprotein; SBP: systolic blood pressure; DBP: diastolic blood pressure; MVPA: moderate-to-vigorous physical activity; P : S ratio: polyunsaturated : saturated fatty acids ratio. ^*∗*^Student's *t*-test; ^†^chi-squared test; ^‡^Fisher's exact test. ^1^*N*=141; ^2^*N*=138. ^a^Adjusted for age, BMI, sedentary time, and diabetes; ^b^adjusted for age, gender, sedentary time, and diabetes; ^c^adjusted for age, gender, sedentary time, BMI, and diabetes; ^d^adjusted for age, gender, sedentary time, and BMI; ^e^adjusted for age, gender, BMI, and diabetes; ^§^values were presented backtransformed.

**Table 2:** Demographic and clinical characteristics of participants according to the *IL6* -174G >C (rs1800795) polymorphism.

Variables	Total	-174G >C polymorphism		*p* value^*∗*^	Adjusted *p* value
	*N*=148	GG (*N*=96)	C carriers (*N*=52)		
*Clinical, anthropometrical, and body composition variables*					
Age (years)	39.61 ± 8.62	39.54 ± 7.96	39.75 ± 9.81	0.889	–
Female/male, *N* (%)	126 (85.1)/22 (14.9)	87 (69.0)/9 (40.9)	39 (31.0)/13 (59.1)	**0.011** ^†^	**0.022** ^a^
BMI (kg/m^2^)	45.97 ± 6.38	45.50 ± 6.28	46.84 ± 6.49	0.221	0.386^b^
Fat mass (kg)^1^	61.24 ± 12.93	60.02 ± 11.92	63.44 ± 14.45	0.130	0.219^b^
Fat-free mass (kg)^1^	57.32 ± 9.00	56.41 ± 8.03	58.95 ± 10.41	0.107	0.983^b^
SBP (mmHg)	128.32 ± 17.92	125.52 ± 16.08	125.44 ± 14.46	0.975	0.198^c^
DBP (mmHg)	85.72 ± 13.69	83.13 ± 11.28	81.00 ± 9.72	0.252	0.059^c^
Diabetes, *N*(%)	41 (27.7)	27 (28.1)	14 (26.9)	0.876^†^	0.331^d^
Hypertension, *N* (%)	96 (4.8)	62 (64.6)	34 (65.4)	0.922^†^	0.549^c^
Dyslipidemia, *N* (%)	117 (79.0)	75 (71.1)	42 (80.8)	0.706^†^	0.319^c^
Log MVPA (min/week)^2^	44.52 ± 61.04	50.77 ± 63.42^§^	32.92 ± 55.11^§^	0.115	**0.024** ^e^
Sedentary time (min/day)^2^	1176.82 ± 83.26	1170.42 ± 84.64	1188.71 ± 80.14	0.216	0.230^e^

*Biochemical parameters*					
Fasting glucose (mg/dL)	110.14 ± 45.34	109.48 ± 42.48	111.35 ± 50.61	0.812	0.609^d^
Fasting glucose range, *N* (%)				0.230	0.174^d^
<100 mg/dL	87 (58.8)	53 (60,9)	34 (39.1)		
≥100 mg/dL	61 (41.2)	43 (70.5)	18 (29.5)		
Fasting insulin (*μ*U/mL)	23.38 ± 14.79	23.49 ± 15.64	23.17 ± 13.22	0.901	0.589^d^
HOMA-IR	6.40 ± 4.90	6.45 ± 5.20	6.32 ± 4.33	0.887	0.464^d^
GHb (%)	6.29 ± 1.44	6.26 ± 1.39	6.34 ± 1.54	0.755	0.792^d^
Total cholesterol (mg/dL)	189.86 ± 37.98	188.82 ± 41.36	191.79 ± 31.06	0.652	0.966^c^
HDL cholesterol (mg/dL)	47.57 ± 11.33	47.83 ± 11.65	47.08 ± 10.81	0.700	0.816^c^
LDL cholesterol (mg/dL)	110.03 ± 35.40	109.57 ± 38.68	110.90 ± 28.50	0.830	0.943^c^
Triglyceride (mg/dL)	161.46 ± 78.11	156.56 ± 73.11	170.50 ± 12.01	0.302	0.629^c^

*Dietary intake*					
Energy (kcal)	1699.44 ± 708.99	1652.17 ± 683.44	1786.71 ± 752.88	0.272	0.906^e^
Proteins (%)	17.36 ± 4.59	16.97 ± 4.32	18.08 ± 5.00	0.163	0.177^e^
Carbohydrates (%)	51.57 ± 8.71	52.23 ± 8.65	50.36 ± 8.78	0.216	0.186^e^
Lipids (%)	27.91 ± 6.52	28.02 ± 6.20	27.71 ± 7.13	0.784	0.557^e^
Saturated (%)	8.43 ± 2.71	8.51 ± 2.69	8.29 ± 2.77	0.630	0.459^e^
Polyunsaturated (%)	4.27 ± 1.66	4.13 ± 1.65	4.53 ± 1.66	0.166	0.156^e^
Monounsaturated (%)	7.61 ± 2.61	7.35 ± 2.54	8.12 ± 2.70	0.107	0.216^e^
P : S ratio	0.56 ± 0.29	0.53 ± 0.30	0.59 ± 0.24	0.272	0.056^e^
Cholesterol (g)	219.39 ± 120.29	208.14 ± 114.74	240.16 ± 128.48	0.122	0.434^e^
Fiber (g)	15.17 ± 8.43	15.14 ± 7.72	15.22 ± 9.67	0.952	0.424^e^

Data are presented as mean ± SD or *N* (%). BMI: body mass index; HOMA-IR: homeostatic model assessment for insulin resistance; HbA1c: hemoglobin A1c; HDL: high-density lipoprotein; LDL: low-density lipoprotein; SBP: systolic blood pressure; DBP: diastolic blood pressure; MVPA: moderate-to-vigorous physical activity; P : S ratio: polyunsaturated : saturated fatty acids ratio. ^*∗*^Student's *t*-test; ^†^chi-squared test. ^1^*N*=140; ^2^*N*=143. ^a^Adjusted for age, BMI, sedentary time, and diabetes; ^b^adjusted for age, gender, sedentary time, and diabetes; ^c^adjusted for age, gender, sedentary time, BMI, and diabetes; ^d^adjusted for age, gender, sedentary time, and BMI; ^e^adjusted for age, gender, BMI, and diabetes; ^§^values were presented backtransformed.

**Table 3:** Association between BMI categories and the genotypes in severely obese patients.

Genotypes	BMI (kg/m^2^)		*p* value^*∗*^	Adjusted *p* value^a^
	35–49.9	≥50		
*PPARG2* Pro12Ala (*N*=146)			0.123	0.154
ProPro (*N*=127)	95 (74.8)	32 (25.20)		
Ala carriers (*N*=19)	11 (57.9)	8 (42.1)		
*IL6* -174G >C (*N*=148)			0.907	0.851
GG (*N*=96)	71 (74.0)	25 (26.0)		
C carriers (*N*=52)	38 (73.1)	14 (26.9)		
Genotype combination (*N*=145)			0.361^†^	0.257
No variants (*N*=78)	59 (75.6)	19 (24.4)		
* PPARG2* variant only (*N*=48)	36 (75.0)	12 (25.0)		
* IL6* variant only (*N*=15)	9 (60.0)	6 (40.0)		
Both variants (*N*=4)	2 (50.0)	2 (50.0)		

Data are presented as *N* (%). BMI: body mass index. ^*∗*^Chi-squared test; ^†^Fisher's exact test; ^a^adjusted for age, gender, sedentary time, and diabetes.

## Data Availability

The data used to support the findings of this study are available from the corresponding author upon request.
